# Gynecological Morbidity in Geriatric Women: Findings From a Cross-Sectional Study in a Tertiary Care Center in Northern Sri Lanka

**DOI:** 10.7759/cureus.85416

**Published:** 2025-06-05

**Authors:** Raguraman Sivalingarajah, Balagobi Balasingam, Powsiga Uruthirakumar, Subaschandren Kumaran, Jeyaluxmy Sivalingam, Shribavan Ganeshamoorthy, Shathana Paramanathan

**Affiliations:** 1 Department of Obstetrics and Gynecology, University of Jaffna, Jaffna, LKA; 2 Department of Surgery, University of Jaffna, Jaffna, LKA; 3 Centre for Digital Epidemiology, University of Jaffna, Jaffna, LKA; 4 Department of Community and Family Medicine, University of Jaffna, Jaffna, LKA

**Keywords:** geriatric women, gynaecological disorders, sri lanka, uterovaginal prolapse, vulval lumps

## Abstract

Background and objective

Gynecological disorders are prevalent among elderly women due to ageing and hormonal changes in the post-reproductive period. However, there is limited evidence in Sri Lanka to guide the provision of optimal care for geriatric women with these conditions. Therefore, this study aimed to describe the gynecological disorders among elderly women.

Methods

A hospital-based descriptive cross-sectional study was carried out among 327 women aged 60 years and above who attended the gynecology and urology clinics at the Teaching Hospital Jaffna, Jaffna, Sri Lanka. An interviewer-administered questionnaire and data extraction sheet were used to collect the data. Analysis was performed using SPSS Version 26.

Results

A total of 327 geriatric women (mean age 71.2 ± 7.5 years) were included, with 63.0% (n=206) residing in rural areas and 72.2% (n=236) financially dependent on their children. The most common gynecological symptoms reported were vulval lumps (63.6%, n=208), back pain (60.6%, n=198), storage urinary symptoms (59.3%, n=194), and vaginal discharge (42.8%, n=140). Uterovaginal prolapse was the most prevalent disorder (62.4%, n=204), followed by cystocele (43.1%, n=141) and rectocele (9.5%, n=31). Postmenopausal bleeding was identified in 11.9% of participants (n=39), of which 7.0% (n=23) were benign and 4.9% (n=16) were malignant. Genital malignancies were recorded in 2.4% (n=8, cervix), 3.1% (n=10, endometrium), 2.8% (n=9, ovary), and 0.3% (n=1, vulva).

Conclusion

This study highlights a high burden of gynecological symptoms and disorders, including pelvic organ prolapse, urinary incontinence, and malignancies, among geriatric women in northern Sri Lanka. The findings underscore the urgent need for age-sensitive health services, improved community screening, and integration of geriatric reproductive health into national health policies.

## Introduction

Globally, the ageing population is rapidly increasing, posing significant challenges to healthcare systems. According to the World Health Organization (WHO), the percentage of individuals aged 60 years and above is projected to nearly double from 12% in 2015 to 22% by 2050 [1]. This demographic shift necessitates the enhancement of healthcare services to address the unique health needs of older adults. Based on the WHO in Asia in 2025, the number of advanced age women would increase from 107 million to 373 million due to increased life expectancy and lifestyle changes such as more active post-menopausal life [2,3].

Geriatric gynecology deals with gynecological pathologies essential in post-menopausal women aged 65 years and above and encompasses a range of conditions, including pelvic organ prolapse, urinary incontinence, postmenopausal bleeding, and various benign and malignant gynecological pathologies [4,5]. These conditions can substantially affect physical, psychological, and social well-being. However, many elderly women do not seek medical care for gynecological symptoms due to several reasons. Factors such as the normalization of symptoms with ageing, financial constraints, limited access to healthcare services, and lack of awareness about treatment options contribute to the underutilization of healthcare services [6-8]. Moreover, societal taboos and embarrassment associated with gynecological issues further prevent elderly women from seeking timely medical attention [9].

Despite the increasing burden of gynecological disorders among geriatric women, there is a paucity of research focusing on the prevalence of these conditions in low- and middle-income countries, including Sri Lanka. Understanding the pattern of gynecological morbidity in this population is crucial for developing targeted interventions and improving healthcare delivery. This study aims to explore the spectrum of gynecological morbidity among geriatric women attending a tertiary care hospital in Sri Lanka.

The findings of this study contribute to the existing knowledge of geriatric gynecological health, providing valuable insights for healthcare policymakers and practitioners to optimize healthcare services for ageing women.

## Materials and methods

This hospital-based descriptive cross-sectional study was conducted at the Gynecological Clinic of the Teaching Hospital, Jaffna, Sri Lanka, from January 2023 to October 2024. The Teaching Hospital Jaffna is a tertiary care hospital providing specialized medical services, including gynecological care, to patients in the Northern Province of Sri Lanka. Elderly women aged 60 years and above attending the Gynecological Clinic of the Teaching Hospital Jaffna for gynecological care were included in this study. A total of 327 participants were included in the study using convenience sampling. Women who met the inclusion criteria were recruited on their clinic days. The inclusion criteria consisted of women aged 60 years and above who attended the gynecological clinic during the study period and provided written informed consent. Details of the variables collected, including sociodemographic data, menopausal status, presenting complaints, and co-morbidities, are provided in the Appendix.

A structured interviewer-administered questionnaire was used to collect data from eligible participants during routine clinic hours. A comprehensive training session was conducted for the female data collector, who had a medical background, to ensure standardized and accurate data collection. The training covered study objectives, ethical considerations, interviewing techniques, and the process of extracting clinical information from medical records. Eligible patients were approached on their clinic days, and the study objectives were explained to them. Written informed consent was obtained before data collection, ensuring strict privacy and confidentiality. Data collection involved face-to-face interviews using a structured questionnaire to obtain information on sociodemographic characteristics, presenting complaints or symptoms, and gynecological disorders. Information on diagnosed gynecological disorders was extracted from the participants' clinic books with their permission.

Data analysis

The collected data were entered into a database and analyzed using SPSS Version 26 (IBM Corp., Armonk, NY). Descriptive analysis was performed, where categorical variables were summarized using frequencies and percentages, and numerical variables were described using measures of central tendency (mean, median) and dispersion (standard deviation, interquartile range).

Ethical consideration

Ethical approval for the study was obtained from the Ethics Review Committee, Faculty of Medicine, University of Jaffna (J/ERC/23/148/NDR/0297). Ethical considerations included obtaining informed written consent from all participants before enrollment, maintaining the confidentiality and anonymity of participants' information, and allowing participants to withdraw from the study at any stage without any consequences.

## Results

In total, 327 patients attending clinics were included, with a mean (SD) age of 71.2 (±7.54) years. Table 1 presents the sociodemographic characteristics of the study participants. The majority (63.0%, n=206) resided in rural areas, and 99.4% (n=325) were Sri Lankan Tamils; 80.1% (n=262) identified as Hindus. Regarding marital status, 60.9% (n=199) were living with a partner. Most participants (71.9%, n=235) had an education level below ordinary level (O/L). Regarding previous occupation, 23.2% (n=76) had been employed, and 76.8% (n=251) were housewives. More than half (55.0%, n=180) belonged to nuclear families, and the primary source of financial support was children (72.2%, n=236), followed by spouses (10.1%, n=33) and pension schemes (9.8%, n=32). Among the women, 7.0% (n=23) had never given birth, and more than half (56.3%, n=184) were multiparous. The mean (SD) of menopausal age was 47.3 (±5.2) years, the majority (87.5%, n=286) had reached menopause naturally, early or surgical menopause was reported by 4.3% (n=14), and only 2.1% (n=7) were using hormonal replacement therapy (HRT).

**Table 1:** Sociodemographic characteristics of elderly women (n=327) O/L, ordinary level

Sociodemographic factors	n (%)
Age (years)	
60-70	172 (52.6)
Above 70	155 (47.4)
Residential area	
Rural	206 (63.0)
Urban	121 (37.0)
Ethnicity	
Sri Lankan Tamil	325 (99.4)
Others	2 (0.6)
Religion	
Hindus	262 (80.1)
Others	65 (19.9)
Marital status	
Living with partner	199 (60.9)
Not living with partner	128 (39.1)
Educational level	
Below O/L	235 (71.9)
O/L and above	92 (28.1)
Previous occupation	
Employed	76 (23.2)
Housewife	251 (76.8)
Type of family	
Nuclear	180 (55.0)
Extended	147 (45.0)
Economic support	
Self-earned	22 (6.7)
Spouse	33 (10.1)
Siblings	22 (6.7)
Children	236 (72.2)
Pension scheme	32 (9.8)
Others	22 (6.7)

Table 2 describes the reported gynecological symptoms among the women. A lump at the vulva was the most common symptom (63.6%, n=208). Among lower urinary tract symptoms, dysuria was present in 33.6% (n=110), voiding symptoms in 34.9% (n=114), and storage symptoms in 59.3% (n=194). Pain-related symptoms included abdominal distension (22.3%, n=73), lower abdominal pain (37.0%, n=121), and back pain (60.6%, n=198). Regarding vulval symptoms, vulval itching was reported by 18.7% (n=61) of participants, while vaginal discharge was noted in 42.8% (n=140). Regarding urinary incontinence, stress, urge, and mixed incontinence were experienced by 28.1% (n=92), 43.1% (n=141), and 19.9% (n=65), respectively.

**Table 2:** Gynaecological symptoms (n=327)

Gynecological symptoms	n (%)
Lump at vulva	208 (63.6)
Lower urinary tract symptoms	
Dysuria	110 (33.6)
Voiding symptoms	114 (34.9)
Storage symptoms	194 (59.3)
Pain symptoms	
Abdominal distension	73 (22.3)
Lower abdominal pain	121 (37.0)
Back pain	198 (60.6)
Vulval symptoms	
Vulval itching	61 (18.7)
Vaginal discharge	140 (42.8)
Urinary incontinence	
Stress incontinence	92 (28.1)
Urge incontinence	141 (43.1)
Mixed incontinence	65 (19.9)

Considering long-term comorbid conditions, hypertension (59.0%, n=193), diabetes mellitus (34.6%, n=113), heart disease (17.1%, n=56), bronchial asthma (12.8%, n=42), thyroid disorders (9.7%, n=32), chronic kidney diseases (6.4%, n=21), anemia (3.4%, n=11), and chronic obstructive pulmonary disease (4.0%, n=13) were identified among women. Additionally, 32.4% (n=106) of them underwent transabdominal hysterectomy, vaginal hysterectomy, appendicectomy, laparotomy, and so on.

The study highlighted a diversity of gynecological disorders among geriatric women; 7.0% (n=23) had benign postmenopausal bleeding compared to 4.9% (n=16) who had malignant postmenopausal bleeding, and 11.3% (n=37) had urogenital infections. The percentage affected by carcinoma in the cervix, endometrium, ovary, and vulva was 2.4% (n=8), 3.1% (n=10), 2.8% (n=9), and 0.3% (n=1), respectively. The prevalence of malignant ovarian masses (3.4%, n=11) was slightly higher than benign ovarian masses (3.1%, n=10). Cystocele, rectocele, and uterovaginal prolapse were observed among 43.1% (n=141), 9.5% (n=31), and 62.4% (n=204), respectively; however, none of them had enterocele (Table 3).

**Table 3:** Gynaecological diseases (n=327)

Gynecological diseases	n (%)
Postmenopausal bleeding	
Benign	23 (7.0)
Malignant	16 (4.9)
Urogenital infections	37 (11.3)
Genital malignancies	
Carcinoma in the cervix	8 (2.4)
Carcinoma in the endometrium	10 (3.1)
Carcinoma in the ovary	9 (2.8)
Carcinoma in the vulva	1 (0.3)
Ovarian mass	
Benign	10 (3.1)
Malignant	11 (3.4)
Cystocele	141 (43.1)
Rectocele	31 (9.5)
Uterovaginal prolapse	204 (62.4)

## Discussion

Our study highlighted that the majority of participants were from rural areas, had low educational attainment, were previously housewives, currently widowed, and financially dependent on their children. There was a broad spectrum of gynecological symptoms and disorders among older women, including vulval lumps, urinary complaints, pain symptoms, vulval itching, and vaginal discharge. Both benign and malignant postmenopausal bleeding, urogenital infections, and malignancies of the cervix, endometrium, ovary, and vulva were identified. Pelvic organ prolapse was frequently observed. Furthermore, chronic conditions such as hypertension, diabetes, and heart disease were common, along with a notable history of gynecological surgeries.

The social circumstances of the study participants underscore the potential economic vulnerability of elderly women, often amplified by reduced healthcare access, limited awareness and understanding of health issues, and available medical services. They are also prone to psychological distress and require social support [10,11].

In the present study, mean (SD) of menopausal age was 47.3 (±5.2) years, which is comparable with women in Asia, ranging from 42.1 to 49.5 years; however, this is slightly less than the average age of a Sri Lankan woman attaining menopause, which is between 49 and 51 years [12]. In our study, only 2.1% of the participants were using HRT. Another study conducted in a gynecology clinic in Sri Lanka found that 38% of women who had undergone hysterectomy and bilateral oophorectomy were using HRT [13]. This discrepancy may be attributed to differences in the study populations, healthcare-seeking behaviors, clinical indications for HRT, or levels of awareness and acceptance of HRT.

The most frequently reported symptom was the presence of a vulval lump, which may reflect a spectrum of conditions ranging from benign cysts to more complex pathologies such as vulvar malignancies. While data on the epidemiology of vulval lesions in South Asia are scarce, studies have shown that such complaints are often underreported due to stigma, lack of awareness, or limited access to care [14]. Lower urinary tract symptoms were also prevalent, affecting a substantial portion of women. The high prevalence observed in our study is comparable to findings from similar settings in Nepal and may be exacerbated by comorbidities such as diabetes, inadequate hydration, and delayed care-seeking behaviors [15]. Urinary incontinence was prevalent in our study population, consistent with findings from a global systematic review, which reported the highest prevalence of urinary incontinence in older women in Asia at 45.1% [16]. This underscores the need for health policymakers to prioritize the development of diagnostic and control measures for urinary incontinence in older women.

The most prevalent condition observed in this cohort was uterovaginal prolapse (62.4%), followed by cystocele (43.1%) and rectocele (9.5%). These findings are consistent with studies conducted in other South Asian settings, where pelvic organ prolapse is a leading cause of gynecological morbidity among parous and postmenopausal women, often due to high parity, poor nutritional status, and limited access to obstetric care during childbirth [17,18]. The burden of prolapse in this population highlights the need for community-level screening and the inclusion of pelvic floor health in reproductive health programs.

A significant proportion of patients also presented with postmenopausal bleeding, with 7.0% due to benign causes and 4.9% due to malignancies. Postmenopausal bleeding is a clinical red flag often associated with endometrial carcinoma or other serious pathology. In our cohort, genital malignancies included carcinoma of the cervix (2.4%), endometrium (3.1%), ovary (2.8%), and vulva (0.3%). These findings align with global cancer burden reports, which indicate that cervical, ovarian, and endometrial cancers are among the most common gynecological malignancies in low-resource settings [19]. The relatively high proportion of malignant ovarian masses (3.4%) also highlights the importance of routine screening and imaging, especially in postmenopausal women, where the index of suspicion for malignancy should be high.

This study has several limitations. Since the study was conducted in a healthcare facility setting, the results may not be generalizable to all older women in the community, particularly those who do not seek care due to financial, cultural, or accessibility barriers. The cross-sectional design limits the ability to draw causal inferences between sociodemographic factors and gynecological conditions. Additionally, some of the data were based on self-reported symptoms, which may be subject to recall bias or underreporting, especially for sensitive conditions such as urinary incontinence or vulval symptoms.

Recommendation

Future studies should focus on identifying and analyzing the factors associated with it. The findings of this study emphasize the importance of strengthening primary and community healthcare systems to include screening and early intervention for chronic gynecological and urological conditions. Awareness programs, caregiver support, and access to diagnostic and specialist care must be enhanced to improve the quality of life of this vulnerable population. Furthermore, the study calls for policies that integrate geriatric reproductive health into national health agendas, ensuring that the specific needs of postmenopausal women are not overlooked in health planning and service delivery.

## Conclusions

This study provides valuable insights into the spectrum of gynecological symptoms and disorders among older women in a Sri Lankan setting, particularly highlighting the interplay between clinical conditions and underlying social determinants of health. The high burden of pelvic organ prolapse, urinary incontinence, vulval symptoms, and gynecological malignancies underscores the pressing need for age-sensitive and accessible women's health services, especially for economically dependent and socially marginalized older women.

## Appendices

A. Sociodemographic Details

Age

Living area: rural / urban

Marital status: married / unmarried / widow / divorced / separated

Ethnicity: Sri Lankan Tamil / Sinhalese / Moor / other

Religion: Hinduism / Christianity / Buddhism / Islam / other

Educational level:

Never been to school

Grade 1-5

Grade 6-10

Up to G.C.E (O/L)

Up to G.C.E (A/L)

Diploma / degree

Previous occupation

Type of family: Nuclear / extended

Source of economic support: self / spouse / siblings / children / pension / others

Parity: Nulliparous / P1 / P2 / P3 / P4 / P5 / >P5

B. Menopause State Details

Age at menopause

Reason: natural / early / surgical

Use of hormonal replacement therapy: Yes/No

C. Chief presenting complaints / symptoms (Yes/No):

Something coming out of vagina

Postmenopausal bleeding

Abdominal distension

Lower abdominal pain

Vaginal discharge

Dysuria

Urinary incontinence

Vulval itching

Vulval growth/ulcer

Back / joint pain

Other (specify)

D. Associated comorbidities (Yes/No):

Hypertension

Diabetes mellitus

Thyroid disorders

Anaemia

Chronic obstructive pulmonary disease

Asthma

Heart disease

Chronic kidney disease

Past surgeries (e.g., hysterectomy, laparotomy)

Other (specify)
